# Editorial: The impacts of the mating system and inbreeding depression in natural plant populations and crop systems

**DOI:** 10.3389/fpls.2024.1528928

**Published:** 2024-12-17

**Authors:** Johanne Brunet, John K. Kelly

**Affiliations:** ^1^ Brunet Research, Madison, WI, United States; ^2^ Department of Ecology and Evolutionary Biology, University of Kansas, Lawrence, KS, United States

**Keywords:** colonization success, deceptive orchids, ecological factors, epigenetics, genetic associations, purging of alleles, soft selection, standing genetic variation

## Introduction

Inbreeding depression (ID) and selfing rate are closely connected. The increase in homozygosity associated with selfing leads to a decrease in fitness and to ID, while ID affects the evolution of selfing. By transmitting an extra allele via self-pollen, selfers have a 3:2 genetic advantage over outcrossers. Based on this simple consideration, ID greater than 0.05 favors complete outcrossing; ID less than 0.5 favors complete selfing.

The Research Topic comprises six papers that present new theoretical or empirical perspectives on different aspects of ID and mating system evolution and their impact in natural populations. Few papers on crop systems were submitted and none were accepted by the journal.

## Theory

Population genetics reveals a mechanism for inbreeding depression through homozygosity, and explores the role of ID in the evolution of plant mating systems. Cheptou reviewed studies on inbreeding depression in plants, giving special emphasis to the interplay between expectations from population genetic theory and empirical data. The author pointed out the weak empirical evidence to support purging, that reduces ID in highly inbred populations. Moreover, the occurrence of high ID in selfers, does not support the predictions of models for the evolution of plant mating systems. In his study, he discusses how epigenetics, in particular DNA methylation, provides a mechanism to explain the impact of ecological factors on ID. Cheptou recommends that future studies of the selective role of ID in natural populations consider the role of epigenetics, as this may help resolve some of the observed discrepancies between theory and empirical data.

Genetic associations between loci have been an important focus of investigation in the evolution of mating systems. In part, this is because selfing can impede the breakdown of linkage disequilibria, which usually occur rapidly with random mating. Xu considered the evolution of genetic associations between selfing rate and the deleterious mutations that cause inbreeding depression. His modeling showed that as the strength of selection against deleterious mutations increases, the association between the selfing rate and the magnitude of inbreeding depression changes from positive to negative. Such associations can significantly alter the evolution of the mean selfing rate in the population. Based on these results, Xu suggests better ways to measure the population-level inbreeding depression in terms of the correlation between individual selfing rates and fitness components.

## Evolution of the selfing syndrome based on standing genetic variation

The evolution of selfing in plant populations is typically associated with a size reduction for traits directly affecting selfing, such as herkogamy (the distance between anthers and stigmas), and traits involved in pollinator attraction such as flower size. The evidence supporting the evolution of the ‘selfing syndrome’ comes mostly from comparisons of species pairs with distinct mating systems. Tusuubira and Kelly experimentally determined the role of standing genetic variation in the evolution of traits associated with the ‘selfing syndrome’. After 10 generations of partial or complete selfing, experimental populations evolved large changes in traits that directly impact both selfing and pollinator attraction. Their results support a rapid response to selection under pollinator loss, a response based on standing genetic variation within a single population.

## Soft selection, inbreeding depression and selfing rate evolution

The discrepancy between theory and data on the importance of purging in eliminating inbreeding depression in selfing populations could be partly explained by soft selection, where the fitness of selfed individuals depends on the frequency and density of selfed vs. outcrossed individuals in the population. Walker and Spigler created purely outcrossed, mixed (50% outcrossed-50% selfed) or purely selfed neighborhoods of the mixed mating biennial *Sabatia angularis* (Gentianaceae) and measured inbreeding depression in competitive ability at different plant densities (doubling between 10 and 640). They observed a statistically significant interaction between neighborhood and density on plant size. Density-dependent effects on plant size were greatest in outcrossed neighborhoods suggesting their superior competitive ability. Variations in size and size inequalities were greatest in mixed relative to homogeneous neighborhoods at the final census, suggesting asymmetric competition. The authors explain how their findings align with the concept of soft selection, which can mitigate the genetic load associated with inbreeding depression and its demographic consequences.

## The role of inbreeding depression and mating system during colonization

Colonizing species often have small, isolated populations that are prone to genetic bottlenecks, genetic drift and inbreeding. A mixed mating system, where both selfing and outcrossing can occur, can improve colonization success. Despite the potential importance of inbreeding depression and mating systems in the colonization process, few studies have examined their impact in invasive species.

Balogh and Barrett estimated the relative fitness of selfed and outcrossed offspring of an invasive plant, *Lythrum salicaria*, purple loosestrife, a partially self-incompatible tristylous species. Plants were grown in the presence and absence of intraspecific competition from selfed or outcrossed neighbors. The cumulative inbreeding depression over four years was 48-68% (depending on the method of estimation for multiplicative fitness), lower than many estimates from outcrossing perennial plant species, possibly as a result of the species’ autotetraploidy. The competition treatment did not consistently alter inbreeding depression, a pattern previously reported in different studies. Outcrossing and heterostyly are maintained in this invasive *L. salicaria* population suggesting strong selection against selfed individuals.

Dudash et al. compared mating system data across the continuum of the colonization process. Outcrossing rate (t), inbreeding coefficient (F), and inbreeding depression (ID) obtained from molecular marker data were compared between several native, naturalized, and invasive populations of *Mimulus guttatus*. The mixed mating system persisted across all populations of *Mimulus guttatus*, irrespective of whether they were native, naturalized or invasive. Population outcrossing rates were higher in native populations relative to naturalized populations, but not in invasive populations. Invasive populations had higher inbreeding depression than native populations, but the relationship between outcrossing rate and inbreeding depression varied across populations making it difficult to predict the role of ID in the colonization process.

## Inbreeding depression in deceptive orchids

Finally, Wróblewska et al. quantified inbreeding depression (ID) in three species of “food-deceptive” orchids by comparing reproductive traits between inbred and outbred plants generated via hand pollination. They reported large differences in the magnitude of ID among these species.

